# Epidemiology of Ventilator-Associated Pneumonia, microbiological diagnostics and the length of antimicrobial treatment in the Polish Intensive Care Units in the years 2013-2015

**DOI:** 10.1186/s12879-018-3212-8

**Published:** 2018-07-06

**Authors:** Michał Wałaszek, Anna Różańska, Marta Zofia Wałaszek, Jadwiga Wójkowska-Mach, The Polish Society of Hospital Infections Team, Joanna Domańska, Grzegorz Dubiel, Joanna Liberda, Agnieszka Misiewska-Kaczur, Marzena Lech

**Affiliations:** 1 Polish Society of Hospital Infections, Kraków, Poland; 2 Department of Microbiology, Jagiellonian University Collegium Medicum, ul. Czysta 18, 31-121 Kraków, Poland; 3 State Higher Vocational School in Tarnów, 33-100 Tarnów, Poland

**Keywords:** Ventilator-associated pneumonia (VAP), Healthcare-associated infections (HAI), Intensive care unit (ICU), Poland, *Acinetobacter baumannii*

## Abstract

**Background:**

Ventilator-associated pneumonia (VAP) is a common nosocomial infection in intensive care units (ICUs). The objective of this study was to describe the epidemiology and microbiology of VAP in Polish ICUs from 2013 to 2015, as well as to understand how these depended on the diagnostic methods used to identify VAP pathogens and the clinical strategy for VAP treatment.

**Methods:**

This observational study was carried out in seven Polish adult ICUs. VAP surveillance was based on the European Healthcare-associated Infections Surveillance Network recommendations and was defined as pneumonia occurring more than 48 h after receiving mechanical ventilation, with symptom onset 3 days or more after the hospital stay. Depending on the microbiological diagnostic method, VAP cases were classified as PNEU-1 (positive quantitative culture from minimally contaminated lower respiratory tract specimen such as broncho-alveolar lavage, protected brush or distal protected aspirate) or other VAP cases.

**Results:**

The incidence of VAP was 8.0% and the incidence density: 12.3/1000 ventilator days. Microbiological diagnosis was made using PNEU-1 in 80 cases (39%); over the study duration, the proportion of cases diagnosed with PNEU-1 increased from 14 to 60% (*p* < 0.001). The predominant etiologic agents causing VAP were Enterobacteriaceae (32.6%) and non-fermenting Gram-negative bacteria (27.6%). The causative microbe varied significantly depending on the diagnostic method: in cases diagnosed using PNEU-1, *Staphylococcus aureus* (21.3%) and *Klebsiella pneumoniae* (12.5%) were the dominant organisms, whereas in other VAP cases, *Acinetobacter baumannii* (23.8%) was commonly observed. The length of antibiotic treatment in cases diagnosed with PNEU-1 was shorter than for other VAP cases (7.2 vs. 9.1 days, *p* < 0.005), as was the duration of hospitalization (49 vs. 51.8 days, *p* < 0.001). Antibiotic resistance was a particular concern for *A.baumannii* isolates, which were highly resistance to imipenem (70.6%) and meropenem or doripenem (52.9%). *K. pneumoniae* isolates demonstrated resistance to ampicillin (90.3%), ceftazidime (71.0%) and third-generation cephalosporins (74.2%).

**Conclusion:**

A change over time was observed in the microbiological diagnostic methods used for patients with VAP. *A. baumannii* was observed mainly in VAP cases diagnosed using substandard methods (non-PNEU-1). The duration of treatment for VAP patients diagnosed properly using PNEU-1 was shorter.

## Background

Intensive care unit (ICU) hospitalizations impose a high risk of acquiring healthcare-associated infection (HAIs), most commonly nosocomial pneumonia (PNEU). In many cases, the patient’s underlying disease and critical condition necessitates invasive procedures and diagnostics, which may contribute unavoidably to the patient’s risk of colonization by the exogenous microbes [1–3]. One of the most common invasive procedures is intubation, and an artificial respiratory tract eliminates the physiological functions (heating, humidification and purification) of the upper respiratory mucosa, thus increasing the risk of ventilator-associated PNEU (VAP) [4–7]. The microorganisms responsible for VAP and their drug resistance vary between individual hospitals wards, between regions of a country and across Europe [8]. Thus, there is a need for local surveillance data, taking into account a detailed analysis of etiologic agents responsible for VAP, which may be highly relevant in implementing local procedures for PNEU prevention [1, 9, 10]. Lower respiratory tract specimens are preferred for microbiological diagnosis of VAP, but invasive sampling may not always be possible. Furthermore, the importance of microbiological diagnosis is underestimated by Polish physicians and it remains underutilized [11, 12], despite the fact that microbiological consult can yield better results from antimicrobial therapy with decreased costs [13].

The main objective of this study was to describe the epidemiology of nosocomial VAP in Polish ICU patients, and to compare these results to the European HAI-Net program. The second aim was to understand the microorganisms responsible for VAP, their drug sensitivity and the interventions required to treat the infections, as well as how these depended on the microbiological diagnostic method.

## Methods

The analysis was carried out in seven general adult ICUs located in southern Poland from 2013 to 2015. The information was collected through hospital participation in a standardized program run based on the European HAI-Net. The Healthcare-Associated Infections Surveillance Network (HAI-Net) is a European network for surveillance of HAIs. The network is coordinated by the European Centre for Disease Prevention and Control (ECDC), an EU agency established in 2005and aimed at strengthening Europe’s defenses against infectious diseases. Participation in HAI-Net is voluntary and confidential for ICUs. In Poland, the coordinator of HAI-Net was the Polish Society of Hospital Infections, a non-governmental organization. Detailed descriptions of data collection systems, study units, epidemiology of bloodstream infections and their microbiology have been previously published elsewhere [14, 15]. All of the units participating in the study were part of non-teaching multi-profile hospitals, with an average number of 414 beds (range: 224–669). The average full time-equivalent nurse per bed ratio was 2.6. Nearly all six of the surveyed units were trauma and medical ICUs without cardiac surgery, neurosurgery or oncology (no immunocompromised, hematological and/or transplant patients); one unit was a surgical ICU. In terms of ICU type and patient characteristics, the study units were typical Polish ICUs. Care bundles were not used for ventilated patients.

The study excluded ICU patients whose hospital stay was shorter than 2 days and patients who showed symptoms of infection within 2 days of admission. The intubation utilization ratio was 0.79.

PNEU was diagnosed and classified based on the uniform definitions issued by the ECDC for all ICUs covered by surveillance. Only cases involving VAP were included in the analysis. VAP was defined according to the ECDC as PNEU (diagnosed using clinical and microbiological criteria) occurring more than 48 h after patients received mechanical ventilation, with symptom onset 3 days or more after the hospital stay [16, 17]. Depending on the microbiological diagnostic method, VAP cases were classified as PNEU-1 (minimally contaminated lower respiratory tract sample with quantitative culture), PNEU-2 (non-protected sample, such as endotracheal aspirate, with quantitative culture), PNEU-3 (alternative microbiological criteria such as positive blood culture), PNEU-4 (sputum bacteriology or non-quantitative), or PNEU-5 (no microbiological documentation) [16, 17].

The following epidemiological measures were used: (i) the cumulative incidence of VAP was calculated by dividing the number of VAP cases by the number of patients admitted to the ICU and multiplying by 100; (i) the density incidence of VAP was calculated by dividing the number of VAP cases by the number of ventilator days (pds) and multiplying by 1000; and (iii) the case fatality rate was calculated by dividing the number of deaths of VAP cases by the total number of VAP cases and multiplied by 100. Statistical analysis of the data was performed using SPSS software (SPSS – Statistical Package for the Social Sciences) STATISTICS 24, Armonk, NY, USA. For statistical analysis of ordinal or dichotomous data, information on the number and percentage of individuals was used. In order to compare differences between the duration of antibiotic treatment and duration of hospital stay, the mean, median (Me), standard deviation (SD), 95% confidence interval (95% CI), minimum and maximum were calculated. For ordinal and nominal variables, Pearson’s chi-square (χ^2^) test was used. Statistical significance was assumed at a level of *p* < 0.05.

### Ethics approval and consent to participate

This study was approved by the Bioethics Committee of Jagiellonian University (approval no. KBET /122.6120.118.2016 from 25.05.2016). All data used in this study were anonymized prior to analysis.

## Results

**Fig. 1:**
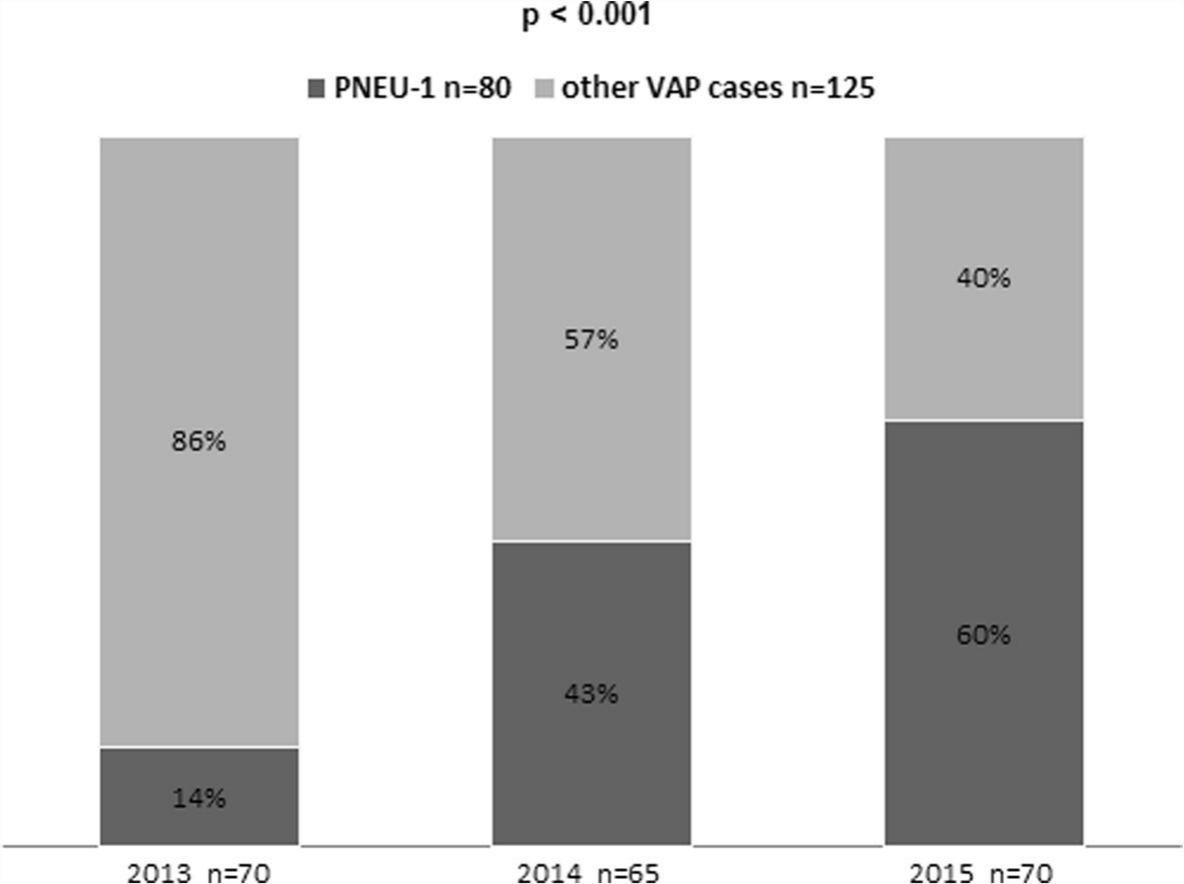
Diagnostic category of ICU-acquired pneumonia, 2013–2015

Over the period analyzed, there were 2547 patients hospitalized for over 48 h in the seven Polish ICUs and 205 VAP cases. The number of patients hospitalized in each ICU ranged from 206 to 628. The cumulative VAP incidence was 8.0% and the incidence density was 12.3/1000pds. Microbiological diagnosis was made in 80 VAP cases (39%) using PNEU-1and over the study duration, the proportion of VAP cases diagnosed using PNEU-1increased from 14 to 60% (*p* < 0.001) (Fig. 1).

The average time from admission to VAP diagnosis was 26 days (SD 91.8; Me 9, 95%CI 13.5–38.7,). The average duration of hospitalization of VAP cases was 51 days (SD 97.1; Me 31, 95% CI 37.2–64.4). The duration of hospitalization was not significantly shorter for VAP cases diagnosed using PNEU-1 (49 days, SD 89.1; Me 31, 95% CI 29.3–69.2) compared with other VAP cases (51.8 days, SD 102.3; Me 31, 95% CI 33.3–70.4,).

The predominant etiologic agents responsible for VAP were Enterobacteriaceae (32.6%) and non-fermenting Gram-negative bacteria (27.6%). The causative microbe varied significantly depending on the diagnostic method. In VAP cases diagnosed using PNEU-1, *Staphylococcus aureus* (21.3%) and *Klebsiella pneumoniae* (21.3%) were the dominant organisms, whereas in other VAP cases, *Acinetobacter baumannii* (30.2%) and *K. pneumoniae* (15.9%) were commonly observed (Table 1).

**Table 1:** Microorganisms isolated in VAP depending on the employed method of taking the material for microbiological diagnostics, Polish ICUs 2013–2015

VAP	PNEU-1^a^	Other VAP cases^b^	Total
Gram-positive cocci n(%)			35 (19.3)
*Staphylococcus aureus*	17 (21.3)	10 (9.9)	27 (16.1)
Coagulase-Negative Staphylococci	0 (0.0)	1 (1.0)	1 (0.6)
*Enterococcus spp.*	1 (1.3)	1 (1.0)	2 (1.2)
*Streptococcus pneumoniae*	4 (5.0)	1 (1.0)	5 (3.0)
Enterobacteriaceae n(%)			59 (32.6)
*Citrobacter spp.*	0 (0.0)	1 (1.0)	1 (0.6)
*Enterobacter spp.*	1 (1.3)	2 (2.0)	3 (1.8)
*Escherichia coli*	5 (6.3)	7 (6.9)	12 (7.1)
*Klebsiella pneumoniae*	17 (21.3)	16 (15.8)	31 (18.5)
*Proteus spp.*	2 (2.5)	4 (4.0)	6 (3.6)
*Seratia spp.*	5 (6.3)	1 (1.0)	6 (3.6)
Non-fermenting Gram-negative bacteria n(%)			50 (27.6)
*Acinetobacter baumannii*	10 (12.5)	24 (23.8)	34 (20.2)
*Pseudomonas aeruginosa*	8 (10.0)	2 (2.0)	10 (6.0)
*Stenotrothomonas maltophilia*	1 (1.3)	1 (1.0)	2 (1.2)
*Hemophilus spp.*	0 (0.0)	4 (4.0)	4 (2.4)
Other n(%)			37 (20.4)
Other bacteria	20 (25.0)	17 (16.8)	20 (22.0)
Total n(%)	80 (100.0)	101 (100.0)	181 (100.0)

*VAP* Ventilator-Associated Pneumonia

^a^PNEU-1: pneumonia documented by invasive diagnostics, minimally contaminated lower respiratory tract sample with quantitative culture

^b^Other VAP cases, documented by: PNEU-2 non-protected sample with quantitative culture; PNEU-3 alternative microbiological criteria; PNEU-4 sputum bacteriology or non-quantitative culture

Antibiotic resistance was a particular concern for *A. baumannii* isolates, which were highly resistant to carbapenems, including imipenem (24 strains, 70.6%) and meropenem or doripenem (18 strains, 52.9%). *K. pneumoniae* isolates were commonly resistant to ampicillin (28 strains, 90.3%), ceftazidime (22 strains, 71.0%) and third-generation cephalosporins (23 strains, 74.2%) (Table 2).

**Table 2:** Antimicrobial resistance in Polish ICUs by VAP, 2013–2015

	Number of isolates	Antibiotic or resistant codes	Non susceptible N (%)
Gram-positive cocci			
*Staphylococcus aureus*	27	MRSA	2 (7.4)
		ampicillin	0 (0)
		glycopeptides	0 (0)
Enterobacteriaceae			
*Escherichia coli*	12	ampicillin	7 (58.3)
		ceftazidime	3 (25.0)
		cefotaxim/ceftriaxone	3 (25.0)
		imipenem	1 (8.3)
		meropenem/doripenem	0 (0)
		ESBL	1 (8.3)
*Klebsiella pneumoniae*	31	ampicillin	28 (90.3)
		ceftazidime	22 (71.0)
		cefotaxim/ceftriaxone	23 (74.2)
		imipenem	0 (0)
		meropenem/doripenem	0 (0)
		ESBL	15 (48.4)
Non-fermenting Gram-negative bacteria			
*Acinetobacter baumannii.*	34	imipenem	24 (70.6)
		meropenem/doripenem	18 (52.9)
		colistin	0 (0.0)
		sulbactam	14 (41.2)
*Pseudomonas aeruginosa*	10	piperacillin	2 (20.0)
		ceftazidime	2 (20.0)
		colistin	0 (0.0)
		imipenem	2 (20.0)
		meropenem/doripenem	2 (20.0)

*ESBL* Extended Spectrum Beta-lactamase, *MRSA* methicillin-resistant Staphylococcus aureus, *VAP* Ventilator-Associated Pneumonia

VAP cases were typically treated using beta-lactams such as third-generation cephalosporins (18.8%), carbapenems (18.1%), and penicillins (16.1%). Subsequent treatments included polymyxins (colistin, 14.1%); aminoglycosides (amikacin, 10.7%) and other drugs (Table 3). The length of antibiotic treatment in VAP cases diagnosed with PNEU-1 was significantly shorter than for other VAP cases (7.2 days vs. 9.1 days, *p* < 0.05) (Table 4).

**Table 3:** Distribution of antimicrobials used for Ventilator-Associated Pneumonia treatment in studied ICUs, 2013–2015, no data: 56 (27%)

Antimicrobial agents	ATC codes		Antibiotic	N (%)
Beta-lactam antibacterials, penicillins (16.1% of all antimicrobials)				24 (100)
Penicillins, with extended spectrum	J01CA	J01CA12	Piperacillin	5 (20.8)
Beta lactamase resistant penicillins	J01CF	J01CF02	Cloxacillin	5 (20.8)
Combinations of penicillins incl. Beta-lactamase inhibitors	J01CR	J01CR02	Amoxicillin and enzyme inhibitor	14 (58.3)
Other beta-lactam antibacterials (37.6% of all antimicrobials)				56 (100)
Second-generation cephalosporins	J01DC	J01DC03	Cefamandole	1 (1.8)
Third-generation cephalosporins	J01DD	J01DD02	Ceftazidime	3 (5.4)
		JO1DD04	Ceftriaxone	4 (7.1)
		JO1DD01	Cefotaxime	21 (37.5)
Carbapenems	J01DH	J01DH02	Meropenem	16 (28.6)
		J01DH51	Imipenem and enzyme inhibitor	11 (19.6)
Other antibacterials (18.8% of all antimicrobials)				28 (100)
Glycopeptide antibacterials	J01XA	J01XA01	Vancomycin	6 (21.4)
		J01XA02	Teicoplanin	1 (3.6)
Polymyxins	J01XB	J01XB01	Colistin	21 (75.0)
Sulfonamides and trimethoprim (4.0% of all antimicrobials)				6 (100)
Combinations of sulfonamides and trimethoprim, incl. Derivatives	J01EE	J01EE01	Sulfamethoxazole and trimethoprim	6 (100.0)
Aminoglycoside antibacterials (10.7% of all antimicrobials)				16 (100)
Aminoglycosides	J01GB06		Amikacin	16 (100.0)
Quinolone antibacterials (9.4% of all antimicrobials)				14 (100)
Quinolone	J01MA	J01MA02	Ciprofloxacin	7 (50.0)
		J01MA12	Levofloxacin	7 (50.0)
Macrolides, lincosamides and streptogramines (1.3% of all antimicrobials)				2 (100)
Lincosamides	J01FF	J01FF01	Clindamycin	2 (100.0)
Antimycotics for systemic use (2.0% of all antimicrobials)				3 (100)
Triazole derivatives	J02AC01		Fluconazole	3 (100.0)
Total				149 (100.0)

**Table 4:** Duration of antibiotic treatment and duration of hospital stay depending on the employed method of taking the material for microbiological diagnostics of VAP, Polish ICUs 2013–2015

	Duration of antibiotic treatment		Length of ICU stay	
	PNEU-1^a^	Other VAP cases^b^	PNEU-1^a^	Other VAP cases^b^
Average (95% CI) (days)	7 (6,8)	9 (8,10)	49 (29,69)	52 (33,70)
Median	7	8	31	31
Standard deviation	3	4	89	102
Minimum	1	3	11	2
Maximum	15	21	781	744
Pearson’s chi-square	(χ^2^ = 8,35, df = 2, *p* < 0.05)		(χ^2^ = 114, df = 69, *p* < 0.001)	

*ICU* Intensive Care Unit, *VAP* Ventilator-Associated Pneumonia

^a^PNEU-1: pneumonia documented by invasive diagnostics, minimally contaminated lower respiratory tract sample with quantitative culture

^b^Other VAP cases, documented by: PNEU-2 non-protected sample with quantitative culture; PNEU-3 alternative microbiological criteria; PNEU-4 sputum bacteriology or non-quantitative culture; PNEU-5 no microbiological documentation

The overall VAP case fatality case rate was 13.6% and fatality case rate was variably associated with infection: PNEU was the direct cause of death in 16 cases (fatality case rate 7.8%), was indirectly connected with death in six cases (2.9%), and was not associated with death in six cases (2.9%). There was no statistically significant difference between fatality case rate and microbiological diagnostic method (PNEU-1 diagnosed invasively vs. other types of VAP) (*p* = 0.08).

## Discussion

According to 2007 data from the European Union, the incidence of VAP was 7% and the incidence density was 8.0/1000 pds [5], slightly lower values than the figures reported in this study. An earlier Polish multicenter study conducted from 2002 to 2003 reported VAP incidence as 6% [18]. However, newer data from 2010 to 2014 and 2015 confirm higher VAP incidence at approximately 9% [3]. The trend toward increasing VAP incidence over the last 10 years may result from improved sensitivity in case detection related to the greater experience of infection control teams, which appeared in Polish hospitals only around the year 2000 [19]. Still, taking into consideration the basic risk factors for VAP (mechanical ventilation and intubation utilization ratio), VAP incidence in Poland was higher than the EU average [5].

No prior studies in Poland addressed the microbiological diagnostic methods that are most commonly employed for VAP, but on the basis of our results, it seems that quantitative cultures from minimally contaminated lower respiratory tract specimens were relatively rarely applied. In the United States, around 60% of VAP cases in medical ICUs are microbiologically diagnosed using state-of-the-art testing methods [20], and in the United Kingdom this figure is around 80% [5]. Fortunately, our data also indicated an increasing proportion over time (14% vs. 60%) of VAP cases diagnosed using PNEU1(based on invasive sampling of material, mainly broncho-alveolar lavage) with a simultaneous decrease in the numbers of VAP cases diagnosed using other methods. These changes show that the awareness of VAP diagnostic methods in ICUs is changing for the better, but also reflects better equipment in the ICUs (including bronchoscopes, making broncho-alveolar lavage possible). Unfortunately, VAP cases without positive microbiology were detected three times more often (14% vs. 5%) in the ICU units studied here than in countries of the European Union [5].

In the Polish ICUs studied here, a large burden of infection by Gram-negative bacilli was documented. A. *baumannii* in particular was responsible for as many as 1/5 cases, and was much more frequently observed than in most European countries [21]. The substantial prevalence of *Acinetobacter* has also been confirmed by other Polish reports [22–24]. *A. baumannii* is not commonly regarded as a major VAP pathogen and because only a minority of VAP microbiological diagnoses were made properly in Polish ICUs, it is difficult to know whether the high prevalence of *A. baumannii* may have resulted from environmental contamination. On the other hand, antimicrobial drug resistance in the tested samples mainly concerned Gram-negative bacilli (e.g., carbapenem-resistant *A. baumannii* and third-generation cephalosporin-resistant *K. pneumoniae*). The data from the ECDC from 2012 onwards point to a significantly lower overall proportion of drug-resistant isolates in Europe. For instance, non-invasive *K. pneumoniae* isolates were, on average, three times less resistant to third-generation cephalosporins than the isolates described in our study (26% vs. 74.2%) [21].

Alp and Damani [25], in their studies of nosocomial infection incidence in low-to-middle income countries, attributed part of the burden of disease to insufficient surveillance of multi-drug resistant organisms. A Human Development Index report from 2015 ranked Poland 36th out of 169 countries, placing Poland among the 25% richest countries in the world. Hence, the high percentage of drug-resistant strains observed in our study is unusual and requires further explanation. Poland underwent a systemic transformation as late in 1989 and, subsequently, legal regulations made it possible to build a nosocomial infection surveillance system. However, this system now seems to be in some extent ineffective [19]. Ider et al. [26] studied countries of the former Eastern bloc that underwent dramatic transformations and noticed certain problems in the nosocomial infection surveillance systems of these countries, including poor commitment, lack of resources, lack of knowledge, and under-reporting of infection statistics. Such situations may result in insufficient prevention and over-treatment of nosocomial infections, which, in turn, can generate antimicrobial resistance.

Our study demonstrates the importance of using appropriate microbiological diagnostic methods in cases of VAP. Our results, showing that the length of antibiotic treatment was significantly shorter when diagnosis was made via quantitative culture from minimally contaminated lower respiratory tracts specimens, are in agreement with those of Fagon et al. who showed that VAP cases diagnosed in this manner require shorter antibiotic treatments and have lower fatality case rate [27]. In a study by Martin-Loeches et al. [28], treatment with an appropriate antibiotic was associated with longer survival in ICUs, and in patients without microbiological confirmation, more antibiotics were required.

The results of our study also showed that patients diagnosed with PNEU-1 stayed in the ICU for a significantly shorter time (about 2 days less) than other VAP patients. Taking into account the costs of ICU hospitalization and the wider circumstances related to healthcare financing, this is a significant benefit both for individual hospitals and for the entire healthcare financing system. In a study of prolonged ICU hospitalization of patients with nosocomial PNEU and its related costs, it was shown that these costs are 40 times higher in Poland than the total costs of outpatient pneumonia [29].

The results of our study indicate the extremely important role of microbiological diagnostic methods in infection control programs. The correct choice of sample material for microbiological diagnosis, as well as optimal methods (as confirmed by a study in a Polish NICU [30]), yields quick and reliable information about the etiological agent and results in a shorter duration of treatment, a shorter length of hospitalization and a lower cost of treatment. At the same time, our results indicate that optimal methods of microbiological diagnosis are insufficiently applied in Poland. This is part of a wider problem in Polish hospitals, which has also been confirmed by studies in different patient population [30–32]. In Polish hospitals, this problem may be related to the lack of physician-microbiologists [33]. Microbiologists or infectious disease physicians are often requested for consultations from other specialty departments with the aim to optimize care for patients with multiple morbidities. Thus, they play a significant role in hospital infection control and antimicrobial stewardship. Studies have shown that microbiological expertise confers significant benefits to other hospital departments including ICUs [22, 34, 35].

This study had some limitations. The first was that it was conducted in a relatively small group of seven ICUs. Most of these ICUs hospitalized patients with similar conditions, but no detailed information was available on comorbidities and no APACHE scores or other health scores were assigned to patients. Another limitation is that, although all study units used the same protocol, there was no external validation of infection detection. Additionally, the protocol did not require gathering detailed data on mechanical ventilation, there were no unique standards of cooperation with microbiology labs, and isolated strains were not banked (and hence no detailed characteristics of etiological agents were available).

## Conclusions

VAP incidence, duration of hospital stay, and fatality case rate in Polish ICUs did not differ from other European countries. Participation in a multicenter infection surveillance system increased the proportion of VAP cases diagnosed using microbiological tests of samples obtained with a method minimalizing the risk of sample contamination.

The reported microbial causes of VAP and their antimicrobial resistance varied significantly depending on the microbiological diagnostic technique. A. *baumannii* isolates were observed primarily in VAP cases diagnosed using substandard microbiological techniques – without positive quantitative culture or corresponding semi-quantitative culture result from minimally-contaminated LRT specimen. The length of antibiotic therapy for VAP cases was significantly shorter when microbiological diagnosis was made using materials collected using a method minimalizing the risk of sample contamination.

## Abbreviations

BAL: Bronchoalveolar lavage
ECDC: Centre for Disease Prevention and Control
ESBL: Extended- spectrum β-lactamase
EU: European Union
HAI: Healthcare-associated infections
HAI-Net: Healthcare-associated Infections Surveillance Network
ICU: Intensive Care Unit
LRI: Lower Respiratory Tract
MDR: Multiple drug resistance organisms
MRSA: Methicillin-resistant *Staphylococcus aureus*
NHSN: National Healthcare Safety Network
NICU: Neonatal Intensive Care Units
PNEU: Pneumonia
PNEU-1: Pneumonia documented by invasive diagnostics, minimally contaminated lower respiratory tract sample with quantitative culture
VAP: Ventilator-associated pneumonia
